# A systematic search for evaluated interventions to increase nursing students’ participation in international scientific conferences: an empty review and implementation implications

**DOI:** 10.1186/s12909-026-08859-8

**Published:** 2026-02-21

**Authors:** Kazumi Kubota, Shiho Samura

**Affiliations:** 1 Research Organization, Shimonoseki City University, 2-1-1 Daigaku-Cho, Shimonoseki, Yamaguchi, 751-8510 Japan; 2 Department of Healthcare Information Management, The University of Tokyo Hospital, Tokyo, Japan; 3 Former Japan International Nursing Student Association, Tokyo, Japan

**Keywords:** Nursing students, International scientific conferences, Systematic review, Empty review, Implementation science, Diffusion of innovations, Theory of planned behavior, Peer mentorship, Language anxiety

## Abstract

**Background:**

International scientific conferences expose nursing students to current evidence, professional identity formation, and global networks, yet participation remains low. We aimed to identify evaluated interventions designed to increase nursing students’ participation in international scientific conferences.

**Methods:**

Following PRISMA 2020, we searched PubMed, CINAHL, Scopus, and Web of Science for English/Japanese records from January 2005 to 31 May 2025. We also examined three International Council of Nurses (ICN) Congress proceedings items authored by the research team as contextual sources. Two reviewers independently screened titles/abstracts. Eligible studies targeted pre-licensure undergraduate nursing students and evaluated strategies intended to increase participation, reporting objective participation outcomes (e.g., abstract submission, registration, attendance, presentation).

**Results:**

Database searches identified 173 records. After removing 58 duplicates, 115 records were screened by title and abstract. All 115 records were excluded at the title/abstract stage; therefore, no eligible evaluative studies were identified (*n =* 0).

**Conclusions:**

No evaluated interventions were identified. This empty review highlights an actionable evaluation gap. Informed by implementation and behavior-change theory and adjacent evidence, we outline implementation implications that can be adopted and monitored using routine indicators (key performance indicators) and iterative improvement cycles (Plan–Do–Study–Act).

**Supplementary Information:**

The online version contains supplementary material available at 10.1186/s12909-026-08859-8.

## Introduction

International scientific conferences, including the International Council of Nurses (ICN) Congress, give nursing students the chance to encounter up‑to‑date scholarship, develop professional identity, and build global networks [1]. Preparing a resilient and internationally engaged nursing workforce is a strategic priority [2]. Yet participation remains low. Across settings, reports point to recurring barriers: late or fragmented information, English language anxiety and presentation apprehension, limited structured peer mentorship, and financial constraints [3–5]. As contextual information, three conference proceeding items authored by the research team (ICN Congress; 2019, 2021, 2023) echo these themes and help contextualize the problem space, but they do not evaluate solutions [6–8].

Closing the gap between interest and action needs attention to both individual decision processes and the organizational conditions that enable sustainable practice. Diffusion theory describes a progression from awareness through interest, evaluation, trial, and adoption, and underlines the role of social systems in sustaining change [9]. In digital environments, information‑seeking and sharing can amplify diffusion after initial awareness (e.g., AISAS: Attention-Interest-Search-Action-Share) [10]. The theory of planned behavior explains how perceived behavioral control and social norms shape intention and behavior [11]. To embed change under real‑world constraints, implementation frameworks such as the Consolidated Framework for Implementation Research (CFIR), RE‑AIM, and Plan-Do-Study-Act (PDSA) provide practical guidance for introduction, evaluation, and maintenance [12–14]. Work on foreign language anxiety shows that avoidance often reflects anxiety rather than ability—a salient point for students considering international scholarly engagement [15]. Sustainable internationalization of curricula also favors integration within existing courses and assessments over ad hoc activities [16].

This study had two objectives. First, we conducted a systematic review to determine whether evaluated interventions exist to increase undergraduate nursing students’ participation in international scientific conferences; no eligible evaluative studies were identified. Second, we propose a minimal set of low‑cost, curriculum‑embedded, student‑led measures that institutions can put in place and steadily improve, with pragmatic metrics for monitoring impact.

## Materials and methods

We conducted a systematic review in line with the PRISMA 2020 Statement [17]. Eligibility criteria, information sources, search strategies, and selection procedures were prespecified. The review protocol was not prospectively registered. To support transparency and reproducibility, we provide the full search strategies, screening forms, and decision rules in the Supplementary Materials. Ethics approval was not required.

### Eligibility criteria

We included studies that: (i) targeted pre‑licensure undergraduate nursing students; (ii) evaluated educational, organizational, financial, or digital strategies intended to increase participation in international scientific conferences; and (iii) reported objective participation outcomes (abstract submission, registration, attendance, presentation). Any comparator was acceptable, including single‑group pre–post designs. We excluded studies focused solely on study abroad, exchanges, or clinical placements without linkage to conferences; studies limited to practicing nurses; and studies reporting only attitudes/intentions without measurable participation endpoints. Conference abstracts and proceedings were treated as contextual (non-eligible) sources where relevant; in practice, we examined three ICN Congress proceedings items authored by the research team as contextual sources [6–8]. Records in English or Japanese from January 2005 through 31 May 2025 were eligible.

### Information sources and search strategy

We searched PubMed, CINAHL, Scopus, and Web of Science Core Collection. In addition, we examined three contextual conference items authored by the research team (ICN Congress proceedings; 2019, 2021, 2023) to describe commonly reported barriers; these items were treated as contextual sources and were not considered eligible evaluation studies. The PubMed strategy was: (("Students, Nursing"[MeSH] OR nursing student*[tiab]) AND (("Congresses as Topic"[MeSH] OR congress*[tiab] OR conference*[tiab] OR "International Council of Nurses"[tiab] OR "Scientific Societies"[MeSH]) AND ("Career Mobility"[MeSH] OR "Career Choice"[MeSH] OR "Education, Nursing"[MeSH] OR "Professional Practice"[MeSH] OR "Professional Competence"[MeSH] OR career*[tiab] OR "professional development"[tiab] OR employ*[tiab])) AND (attend*[tiab] OR participat*[tiab] OR present*[tiab])).

Across all databases, the searches were finalized on 11 October 2025 and were rerun on 29 January 2026 during peer review to support reproducibility; the prespecified end-date limit (31 May 2025) was retained. We applied the end-date limit as 31 May 2025 based on the earliest online publication date (e.g., Epub/online ahead of print) where applicable; records with online publication dates after 31 May 2025 were excluded.

We adapted strategies to each database’s syntax and controlled vocabulary. Full database-specific search strategies are provided in Supplementary File S1.

### Selection process and planned synthesis

Two reviewers independently screened titles and abstracts against the eligibility criteria, resolving disagreements by discussion. Full texts would have been retrieved and assessed for eligibility if potentially eligible records had been identified at screening. Had eligible studies been found, we planned independent data extraction and risk‑of‑bias assessment using ROBINS‑I for nonrandomized designs and appropriate tools for qualitative studies, with tabular/graphical synthesis and, where feasible, meta‑analysis. Given the empty yield, evidence synthesis and risk‑of‑bias assessment were not applicable; we report screening outcomes narratively. Duplicate records were removed using DOI as the primary identifier and PMID as the secondary identifier. Records without DOI/PMID were not deduplicated based on title similarity alone.

## Results

Database searches identified 173 records (PubMed, *n =* 32; Web of Science, *n =* 22; CINAHL, *n =* 52; Scopus, *n =* 67). After removing 58 duplicate records before screening, 115 records were screened by title and abstract. All 115 records were excluded at the title/abstract stage because none evaluated an intervention aimed at increasing participation in international scientific conferences, as prespecified; therefore, no studies were included in the review.

Frequent reasons for exclusion at screening were ineligible populations (e.g., practicing nurses or mixed professional samples without extractable student data), a focus on study abroad, exchanges, or clinical placements rather than scientific conferences, and outcomes limited to attitudes/intentions without measurable participation endpoints. As contextual information, three ICN Congress proceeding items authored by the research team (2019, 2021, 2023) described recurring barriers, including late or fragmented information, English language anxiety, limited peer support, and financial burden [6–8].

## Discussion

This empty review suggests that evaluated interventions to increase undergraduate nursing students’ participation in international conferences have not been reported. The absence of evidence is not evidence of absence, but it does highlight an actionable gap. Institutions already have plausible levers and infrastructure to start making progress while building practice‑based evidence.

To support practical prioritization under routine constraints, we focus on components that can be implemented with minimal additional resources and iteratively refined in routine practice. Drawing on diffusion and behavior theory, implementation science, and work on language anxiety and peer support, we outline a minimal package designed for routine implementation and maintenance.

### Operational feasibility and maintenance

A typical setup assigns one academic lead (or program coordinator) to keep timelines and quality on track—about 1–2 h per week during peak periods (before deadlines) and under an hour otherwise. Automated bilingual announcements can be templated and scheduled in the LMS using a rolling annual calendar; updating dates and links takes ~ 30 min per cycle. Micro–English‑for‑conference workshops fit within first‑year orientation or skills courses as five 60–90‑minute sessions led by an ESP instructor or trained faculty; materials can be reused each year. Student‑led peer mentorship can run as monthly 30‑minute huddles with light faculty oversight.

A consolidated micro‑grants/fee‑waiver webpage can be maintained by administrative staff with a quick quarterly refresh. These choices spread workload and support maintainability despite turnover and budget constraints.

### Equity‑focused implementation and monitoring

To reduce opportunity gaps tied to information access, English anxiety, peer support, and financial burden, announcements and micro‑workshops should sit within required first‑year courses and be repeated at varied times, with hybrid options for commuters and working students. Routine indicators can track: (i) timely awareness before deadlines (overall and by year/commuting/working status); (ii) abstract submission, registration, and presentation rates by household financial hardship and self‑reported English anxiety; (iii) mentee retention/progression, including first‑generation students; and (iv) median out‑of‑pocket costs pre/post micro‑grants/fee waivers. Programs can set simple targets to narrow gaps over time and use PDSA to adjust messaging, workshop sequencing, or funding criteria when disparities persist. RE‑AIM helps capture reach, adoption, fidelity/cost, and maintenance across cohorts [13, 14].

### Strengths and limitations

Strengths include comprehensive searches across four databases over two decades, prespecified and transparent eligibility criteria, and dual independent screening. The main limitation is that no evaluative studies were found; empty yield may reflect unindexed or internal evaluations not publicly available. Still, the empty result is informative: it points to a neglected evaluation space where pragmatic, low‑risk steps can proceed while cumulative evidence is built through routine monitoring and iterative refinement. Future work should test components individually and as a bundle, with mixed‑methods process evaluations to understand mechanisms (anxiety reduction, navigational know‑how, social reinforcement). Agreement on a core outcome set—timely awareness, submission/registration rates, attendance/presentation, changes in English anxiety and self‑efficacy, and cost per participating student—would improve comparability.

## Conclusions

We found no eligible evaluative studies of interventions designed to increase undergraduate nursing students’ participation in international scientific conferences. However, commonly reported barriers appear consistent and potentially modifiable.　A minimal package—early automated bilingual announcements, short micro–English‑for‑conference workshops, structured student‑led mentorship, and a consolidated micro‑grants/fee‑waivers page—can be implemented now using existing infrastructure, with simple indicators to track reach and impact. Iterating through PDSA cycles can progressively strengthen effectiveness and maintainability. As institutions put it into practice and iterate, routine monitoring can help build the practice‑based evidence that has been missing in this area.

## Supplementary Information

Supplementary Material 1.

Supplementary Material 2.

## Data Availability

Data availability No primary datasets were generated or analyzed. Search strategies, the screening log, and the PRISMA source file are available in the Supplementary Materials.
