# Supplementary material for: A systematic search for evaluated interventions to increase nursing students’ participation in international scientific conferences: an empty review and implementation implications
Source: BMC Med Educ. 2026 Feb 21;26:505. doi: 10.1186/s12909-026-08859-8 (PMC13032610; doi:10.1186/s12909-026-08859-8)
Supplement: Supplementary file 1 — Supplementary Material 1. [file 12909_2026_8859_MOESM1_ESM.pdf]

Supplementary File S1. Search strategies, contextual sources, and decision rules

Date searched (all databases): 29 January 2026
Prespecified end-date limit: 31 May 2025 (earliest online publication date where applicable)
Time frame: 01 January 2005 – 31 May 2025
Languages: English or Japanese

Contents

S1-1. Database search strategies (PubMed, Web of Science Core Collection, Scopus, CINAHL/EBSCOhost)
S1-2. Contextual sources (ICN Congress proceedings authored by the research team)
S1-3. Prespecified decision rules (eligibility, deduplication, end-date handling)

S1-1. Database search strategies

(1) PubMed

- **Platform:** PubMed
- **Date searched:** 29 January 2026
- **Language:** English or Japanese
- **Time frame:** 01 Jan 2005 – 31 May 2025
- **Search string (as run):**

(("Students, Nursing"[MeSH] OR nursing student*[tiab]) AND (("Congresses as Topic"[MeSH] OR congress*[tiab] OR conference*[tiab] OR "International Council of Nurses"[tiab] OR "Scientific Societies"[MeSH]) AND ("Career Mobility"[MeSH] OR "Career Choice"[MeSH] OR "Education, Nursing"[MeSH] OR "Professional Practice"[MeSH] OR "Professional Competence"[MeSH] OR career*[tiab] OR "professional development"[tiab] OR employ*[tiab])) AND (attend*[tiab] OR participat*[tiab] OR present*[tiab]))

- **Records exported for screening: n = 32**

(2) Web of Science Core Collection

- **Platform:** Web of Science Core Collection
- **Field searched:** Topic (TS)
- **Date searched:** 29 January 2026
- **Refine applied:** Publication years 2005–2025; Languages English and Japanese
- **Search query (as run):**

```
TS=(
  ("nursing student*" OR "undergraduate nursing" OR prelicensure)
  AND
  (
```

(
(international* OR global OR "world congress" OR "international conference*")
AND (conference* OR congress*)
)
OR
("International Council of Nurses" OR ICN)
)
AND
(attend* OR participat* OR register* OR registration OR submit* OR "abstract submission" OR presentat* OR poster OR "oral presentation")
)

- **End-date handling (31 May 2025):** Web of Science does not allow consistent day-level end-date filtering using the standard "Publication Years" refine option. Therefore, records from year 2025 were manually checked, and items dated on or after 01 June 2025 were excluded based on available online/publication date information.
- **Records exported for screening: n = 22**

(3) Scopus

- **Platform:** Scopus
- **Field searched:** Title/Abstract/Keywords (TITLE-ABS-KEY)
- **Date searched:** 29 January 2026
- **Limits applied:** PUBYEAR > 2004 AND PUBYEAR < 2026; Languages English and Japanese
- **Search query (as run):**

TITLE-ABS-KEY(
("nursing student*" OR "undergraduate nursing" OR prelicensure)
AND
(
(
(international* OR global OR "world congress" OR "international conference*")
AND (conference* OR congress*)
)
OR
("International Council of Nurses" OR ICN)
)
AND
(attend* OR participat* OR register* OR registration OR submit* OR "abstract submission"

OR presentat* OR poster OR "oral presentation")
)
AND (PUBYEAR > 2004 AND PUBYEAR < 2026)

- **End-date handling (31 May 2025):** Scopus does not always allow consistent day-level end-date filtering across all records. Therefore, records from year 2025 were manually checked using available date fields (e.g., cover date/publication date), and items dated on or after 01 June 2025 were excluded.
- **Records exported for screening: n = 67**

(4) CINAHL (EBSCOhost)

- **Platform:** CINAHL Plus with Full Text (EBSCOhost)
- **Fields searched:** Subject Headings (MH), Title (TI), Abstract (AB)
- **Date searched:** 29 January 2026
- **Language limits applied:** English or Japanese
- **Search approach:** Search History sets were created and combined with AND.

S1 (population) (as run):
(MH "Students, Nursing+") OR TI ("nursing student*" OR "undergraduate nursing" OR prelicensure) OR AB ("nursing student*" OR "undergraduate nursing" OR prelicensure)
S2 (international scientific conference concepts) (as run):
(
  (
    (TI (international* OR global OR "world congress" OR "international conference*")
      OR AB (international* OR global OR "world congress" OR "international conference*"))
    AND
    (TI (conference* OR congress*) OR AB (conference* OR congress*))
)
  OR
  (TI ("International Council of Nurses" OR ICN) OR AB ("International Council of Nurses" OR ICN))
)
S3 (participation behaviors) (as run):
TI (attend* OR participat* OR register* OR registration OR submit* OR "abstract submission" OR presentat* OR poster OR "oral presentation")
OR
AB (attend* OR participat* OR register* OR registration OR submit* OR "abstract submission" OR presentat* OR poster OR "oral presentation")
Combined set used for export: S1 AND S2 AND S3

- **Record counts and end-date handling (31 May 2025):**
  - ✓ Records retrieved before limits: **n = 55**
  - ✓ After applying language limits (English/Japanese): **n = 53**
  - ✓ Manual end-date check identified one item dated after 31 May 2025 (August 2025), which was excluded: **n = 1**
  - ✓ **Records exported for screening: n = 52**

S1-2. Contextual sources (ICN Congress proceedings authored by the research team)

These items were examined to describe commonly reported barriers and were **not** considered eligible evaluative intervention studies.

1. Kozera Y, Misawa A, Kido M, Kubota K, Kanai-Pak M. Investigating obstacles of Japanese nursing students participating in the International Council of Nurses Congress. *In:* Proceedings of the International Council of Nurses Congress; 2019 Jun 27–Jul 1; Singapore.
2. Samura S, Furuie A, Hisamatsu H, Kubota K. Obstacles to participation in the ICN Congress for Japanese nursing students. *In:* Proceedings of the International Council of Nurses Congress; 2021 Nov; Virtual.
3. Samura S, Kubota K. Factor analysis of behavioral stage of Japanese nursing students—obstacles to attending the ICN Congress and providing information to students in lower grades. *In:* Proceedings of the International Council of Nurses Congress; 2023 Jul; Montreal, Canada.

S1-3. Prespecified decision rules

Eligibility decision rules

- **Population:** pre-licensure undergraduate nursing students
- **Intervention:** educational, organizational, financial, or digital strategies intended to increase participation in international scientific conferences
- **Outcomes:** objective participation outcomes (abstract submission, registration, attendance, presentation)
- **Exclusions:** study abroad/exchange/clinical placement without linkage to conferences; practicing nurses only; outcomes limited to attitudes/intentions; non-evaluative reports

Deduplication decision rules

- Duplicate records were removed using DOI (primary) and PMID (secondary).
- Records without DOI/PMID were not deduplicated based on title similarity alone.

End-date handling rule

- The end-date limit was 31 May 2025, based on the earliest online publication date (e.g., Epub/online ahead of print) where applicable; records with online publication dates after 31 May 2025 were excluded.

(End)
