# Supplementary material for: A systematic search for evaluated interventions to increase nursing students’ participation in international scientific conferences: an empty review and implementation implications
Source: BMC Med Educ. 2026 Feb 21;26:505. doi: 10.1186/s12909-026-08859-8 (PMC13032610; doi:10.1186/s12909-026-08859-8)
Supplement: Supplementary file 2 — Supplementary Material 2. [file 12909_2026_8859_MOESM2_ESM.pdf]

Figure 2. PRISMA 2020 flow diagram of the study selection process

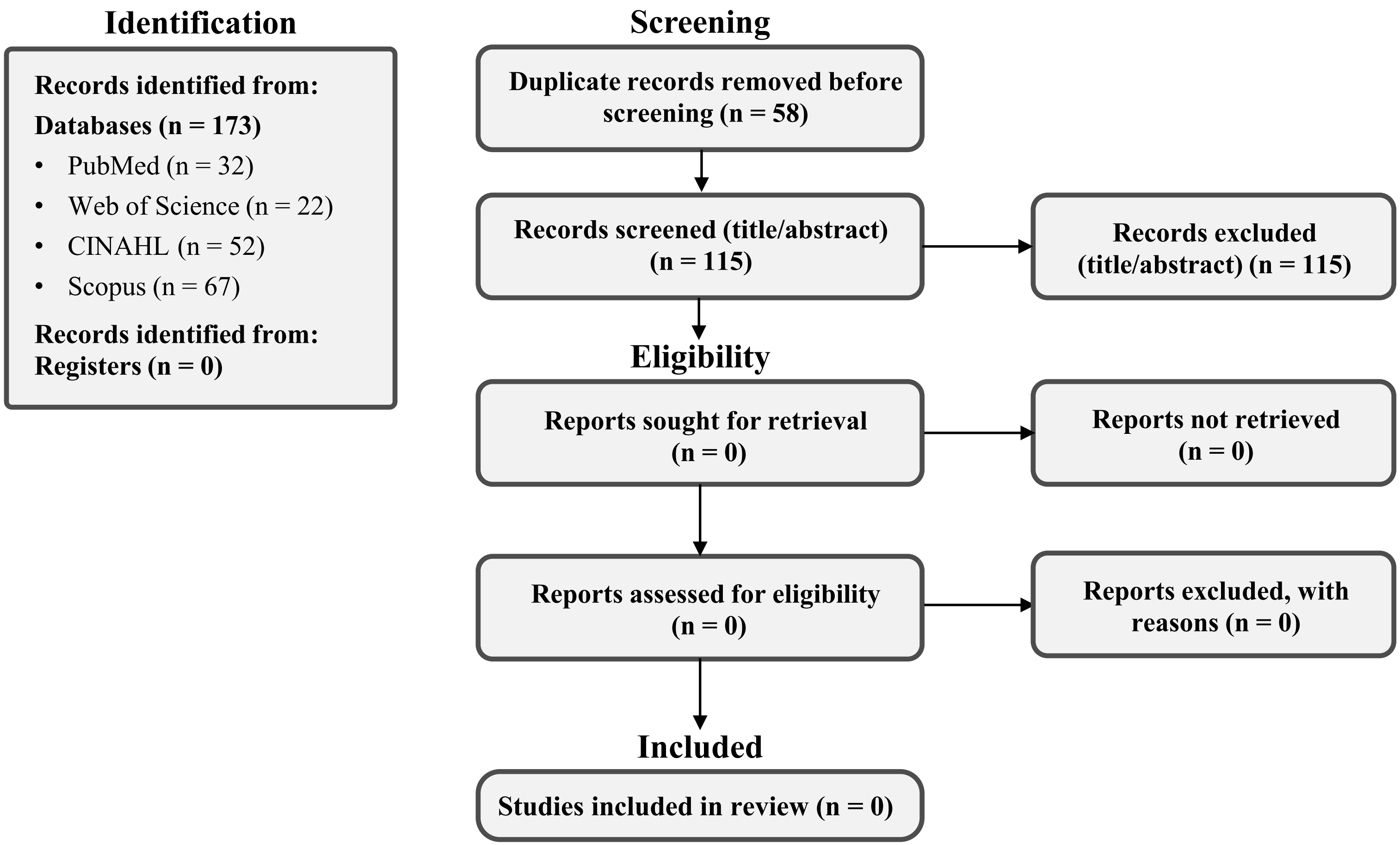

No studies met the inclusion criteria.
